# Overall survival nomogram and relapse-related factors of clear cell sarcoma of the kidney: A study based on published patients

**DOI:** 10.3389/fped.2022.943141

**Published:** 2022-09-16

**Authors:** Yuan Zhang, Qian Chu, Yue Ma, Chunshu Miao, Juan-juan Diao

**Affiliations:** ^1^College of Traditional Chinese Medicine, Shandong University of Traditional Chinese Medicine, Jinan, China; ^2^Department of Pediatrics, Affiliated Hospital of Shandong University of Traditional Chinese Medicine, Jinan, China; ^3^Department of Preventive Treatment, District Second Hospital of Qingdao Huangdao of Traditional Chinese Medicine, Qingdao, China; ^4^Guang'an men Hospital, China Academy of Chinese Medical Sciences, Beijing, China

**Keywords:** clear cell sarcoma of the kidney, nomogram, relapse, prognosis, predictor

## Abstract

**Background:**

Rarity limits the breadth of study on clear cell sarcoma of the kidney (CCSK). There is currently no predictive model that quantifies the overall survival (OS) of CCSK and a few large sample-based analysis of relapse-related factors.

**Methods:**

Patients were collected both from the Surveillance, Epidemiology, and End Results (SEER) database and case report articles extracted from the global online document database to form 2 groups. The first was the OS group, which was used to build and verify the nomogram for predicting the OS of CCSK. Independent predictors of OS were screened by Cox regression analysis to develop the nomogram. Nomogram accuracy was assessed by C-index, receiver operating characteristic (ROC), calibration, and decision curve analysis (DCA) curves. In addition, the difference in OS between receiving radiotherapy or not in stage I patients was analyzed by the Chi-square test. The second was the relapse group, which was used to analyze the relapse-related factors by Cox regression analysis and the Kaplan–Meier method with the log-rank test.

**Result:**

256 patients were included in the OS group. The stage, chemotherapy, and radiotherapy were independent OS-related factors of CCSK, and the nomogram for predicting the OS of CCSK was established based on them. The results of the C-index, ROC, calibration, and DCA curves showed that the nomogram has good discrimination, accuracy, and clinical profitability. The Chi-squared test showed no significant difference in OS with receiving radiotherapy or not in stage I patients. The relapse group included 153 patients, of which 60 relapsed. The univariate Cox regression analysis showed no correlation between radiotherapy and relapse. The multivariate Cox regression analysis showed that stage and surgery/chemotherapy sequence were the independent factors for relapse. The log-rank test of seven chemotherapeutic drugs showed that etoposide (E), cyclophosphamide (C), vincristine (V), and doxorubicin (D) (all *P* < 0.05) had significant differences in preventing relapse, and then drew the relapse-free survival curves of these four drugs.

**Conclusion:**

Our nomogram accurately quantified the OS of CCSK. There was no significant difference in OS between receiving radiotherapy or not in stage I patients. Stage, surgery/chemotherapy sequence, and the use of ECVD were relapse-related factors. Radiotherapy had no significant contribution to preventing relapse.

## Introduction

Clear cell sarcoma of the kidney (CCSK) is a rare pediatric malignant tumor most common in children aged 2–3 years old and accounting for only 3–5% of children's renal tumors (1–3). The clinical features and therapeutic regimen of CCSK which is similar to high-risk nephroblastoma have been systematically studied by the North American National Wilms' Tumor Study Group (NWTSG) and the European International Society of Pediatric Oncology (SIOP) (4–9). The rarity of the tumor limits the study breadth of CCSK, so the prognosis prediction is still poorly understood. In recent years, several studies have illustrated the high correlation between partial gene aberrations and CCSK occurrence at the molecular level, which can be used for the diagnosis of CCSK, such as BCOR-CCNB3 fusion and EGFR mutation (10–13). However, these distortions have not been identified as a prediction of prognosis (14). NWTSG trials 5 (NWTS-5) study showed that age, stage, use of doxorubicin, and necrosis were related risk factors of tumor apoptosis (2). However, it does not specifically quantify the relevance of these factors to the patient's survival. As far as we know, there is currently no predictive model to accurately quantify the overall survival (OS) of CCSK to guide clinical decision-making. Nomogram is a model that provides an accurate prediction of endpoint events with an easy-to-use and efficient interface (15). It is currently being used widely in the diagnosis of tumors and other diseases, which meet our needs.

A combined SIOP and Associazione Italiana Ematologia Oncologia Pediatrica (AIEOP) study showed that relapse occurs in about 16% of patients (5). Several follow-up clinical studies on relapse of CCSK showed that relapse patients who receive only primary treatment with resection + chemotherapy + radiotherapy exhibit a poor prognosis due to the absence of effective treatment standards for relapsed CCSK (16–18). These were mostly single-center studies with a small sample size and no analysis of relapse-related factors. Considering the limitation of the existing treatment, exploring how to avoid relapse is valuable. At present, little research has been done on this topic.

In this study, we obtained patients indirectly from the global online document database and the Surveillance, Epidemiology, and End Results (SEER) database. Two large sample groups were formed to develop nomograms for predicting the OS of CCSK patients with an initial diagnosis. And to analyze relapse-related factors for helping clinicians make more forward-looking clinical decisions.

## Materials and methods

### Data sources and groups

The data sources of this study consist of two parts. Firstly, we used the keywords “Clear cell sarcoma of the kidney” and “Renal clear cell sarcoma” in PubMed, Embase, and China Knowledge Network (CNKI) databases, starting from 1990 to 10 March 2022, and no language restrictions to research articles which published single or multiple cases under 18-years-old with a clear pathological diagnosis of CCSK. The following variables of cases were collected: (i) age at diagnosis, sex; (ii) the earliest clinical symptoms and signs; (iv) primary site, laterality, stage, distant metastases (DM); (v) Surgery (or not), radiotherapy and chemotherapy (or not), surgery/chemotherapy sequence (relative order of chemotherapy and surgery), chemotherapy drugs; (vi) relapse (or not), time of relapse, location of relapse, overall survival, outcome; and (vii) special records. In the study, clinical features and location of relapse were counted using simple summary terms. It is worth noting that the foundation data of constructing a nomogram requires that the information of patients must be specific to the individual level to form a matrix. Hence, some large-sample clinical studies that report the results from a macro perspective could not be included in this study.

Secondly, we obtained cases with CCSK in SEER^*^stat v8.3.9.2 1975–2018 database. The selection statement was the International Classification of Diseases for Oncology, Third Edition (ICD-O-3) histology codes of 8964/3, and age <18. The collected variables included age at diagnosis, sex, the primary site of the tumor, extension, laterality, regional nodes positive, surgery, radiotherapy, chemotherapy, tumor sequence number, cause of death, vital status, and survival months. Calculation of NWTS-5 stage based on the information of regional nodes positive, tumor extension. Limited by incomplete SEER database records, this subset of cases did not contain information of multidisciplinary therapy and relapse.

**Figure 1:**
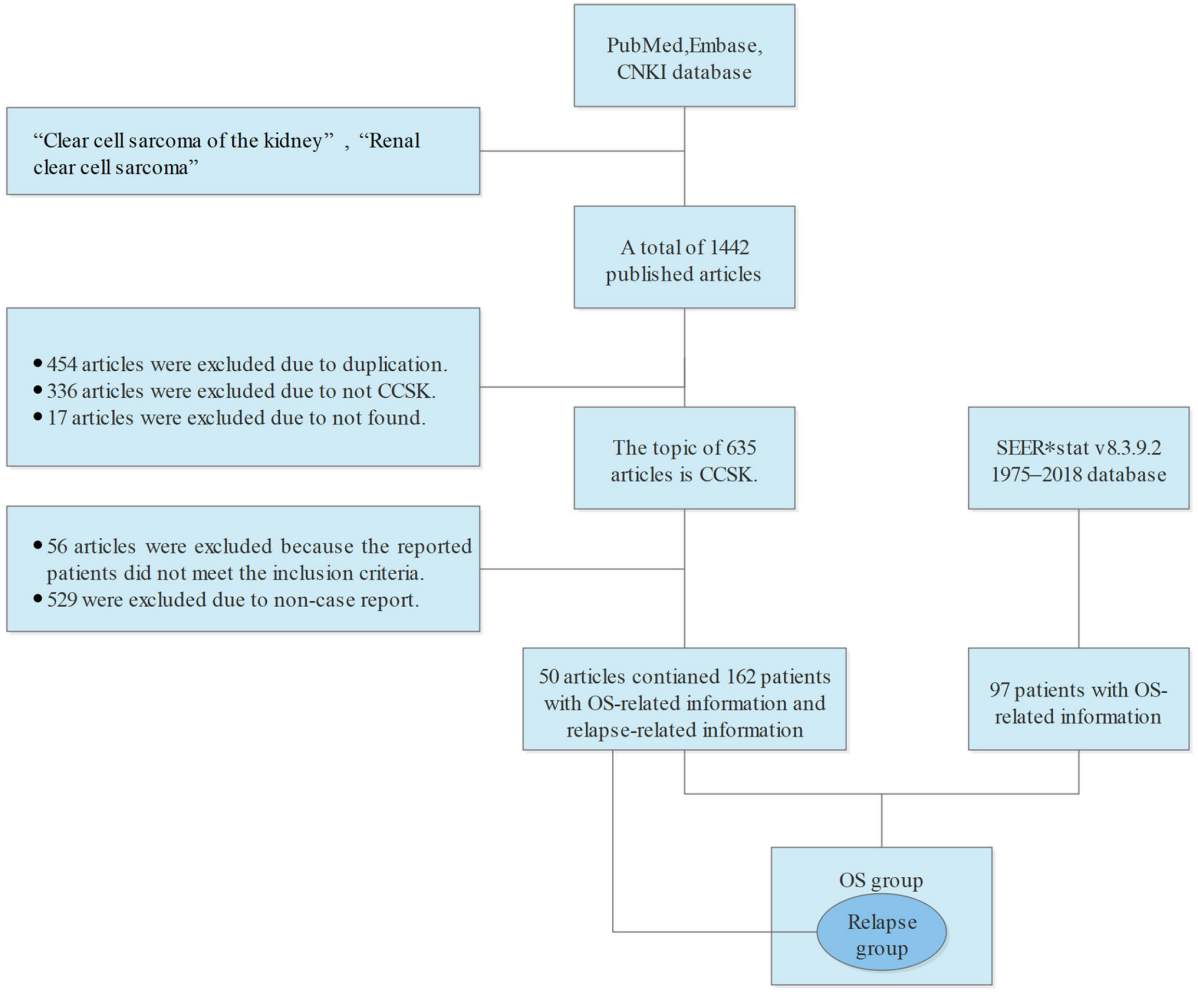
Flow chart of obtaining data and filtering queue.

The information of all variables obtained by the two methods was aggregated to form an OS group which was used to select independent OS-related factors and developed a nomogram with accurate prediction ability for OS of CCSK. Inclusion criteria were each patient's individual variables information (sex, age at diagnosis, laterality, stage, radiotherapy, and chemotherapy) were all available. The exclusion criteria were as follows: (i) CCSK was not the first primary malignant; (ii) Perioperative death. The exact process of obtaining data and screening the groups is shown in the flow chart (Figure 1).

The second was the relapse group, which was used to explore the relapse-related factors. Patients in the OS group with complete information including relapse (or not), surgery/chemotherapy sequence, and use of chemotherapy drugs were extracted to form the relapse group. All the required information was obtained from the global online document because SEER did not record relapse information.

### Nomogram construction and validation in the OS group

R (Version 3.6.1) Software was used for statistical analysis in this study. *P*-value < 0.05 (two-sided) was considered statistically significant. Among all variables, measurement data were presented as mean ± variance. The one-sample K–S test was used to determine whether age conformed to a skewed distribution. The difference in OS between receiving radiotherapy or not in stage I patients was analyzed by Chi-square test.

In the OS group, to increase the layers of verification, patients were randomly divided into a training set (TS) (*n* = 181) and a validation set (VS) (*n* = 78) in a ratio of 7:3. The TS was used to develop the nomogram and the VS was used to validate the accuracy and clinical profitability of the nomogram. The Chi-square test or Fisher's exact test was used to compare the statistical differences of variables between the two groups. The univariate Cox regression analysis was utilized to determine significant OS-related factors. Factors with *P* < 0.05 were included in the multivariable Cox regression analysis to identify the independent predictors which were included in the model for predicting the OS of CCSK and visualized as a nomogram.

We calculated the concordance index (C-index) to assess model discrimination. The nomogram time-dependent ROC curves, calibration curves, and decision curve analysis (DCA) for 12, 36, and 60 months were plotted separately to evaluate the accuracy and clinical profitability of the nomogram. After recording the score corresponding to each predictor in the nomogram, risk stratification was performed by calculating the total score of all patients in the OS group. X-tail Software was used to calculate the best cut-off points for low-risk and high-risk groups. The difference in survival between the two risk groups was demonstrated by Kaplan–Meier (K–M) survival curves with the Log-rank test.

### Selection of relapse-related factors in the relapse group

In the relapse group, we aggregated all variable information to derive relapse characteristics. The univariate Cox regression analysis was utilized to determine significant univariate relapse-related factors with *P* < 0.05. These factors were then screened using multivariate Cox regression analysis for independent relapse-related factors with *P* < 0.05.

Due to differences in the year of treatment and region, patients in the relapse group adopted more than a dozen chemotherapy regimens which could not be unified into a restricted number of regimen options. Hence, we explored the relationship between chemotherapy regimens and relapse from the use of chemotherapy drugs. In order to observe the difference of each chemotherapy drug in preventing relapse, the relapse-free survival curves of chemotherapy drugs were plotted separately by the K–M method, and the differences between the curves were evaluated by the log-rank test.

## Results

### Clinical characteristics in OS group

A total of 259 eligible patients were included in the OS group consisting of 97 patients from the SEER database and 162 patients reported in 50 articles selected from 1442 articles. The articles that were included are shown in Table 1. Information on variables is detailed in Table 2. It was found that the prevalence of CCSK was significantly higher in males than females, with a male-to-female ratio of 2.4:1 (183:76). The tumor's primary renal tendency was homogeneous, left: right = 1.07:1 (134:125). The mean age at initial diagnosis was 2.85 ± 2.19 years, and its normality test showed a left-skewed distribution, with the highest age of onset being 11 years.

**Table 1:** List of articles that were included.

**Author**	**Year**	**Patients included**	**Country**	**Title**
Khalil, R. M.	1993	1	USA	Clear cell sarcoma of the kidney. A case report
Sahjpaul, R. L.	1993	1	UK	Brain metastasis from clear cell sarcoma of the kidney–a case report and review of the literature
Kusumakumary, P.	1997	1	India	Late recurrence of clear cell sarcoma of the kidney
Yumura-Yagi, K.	1998	2	Japan	Successful double autografts for patients with relapsed clear cell sarcoma of the kidney
Parikh, S. H.	1998	1	USA	Clear cell sarcoma of the kidney: an unusual presentation and review of the literature
Yumura-Yagi, K.	1998	2	Japan	Successful double autografts for patients with relapsed clear cell sarcoma of the kidney
D'Antiga, L.	2001	1	UK	Veno-occlusive disease with multi-organ involvement following actinomycin-D
Dundar, E.	2001	1	Turkey	Cerebellar metastasis from clear cell sarcoma of the kidney. A case report with immunohistochemistry
Mazzoleni, S.	2003	1	Italy	Clear cell sarcoma of the kidney in a newborn
Wu, X. R.	2003	3	China	Renal clear cell sarcoma in children:3 cases report and review of literature
Xiong, Z. H.	2003	1	China	Clear cell sarcoma of kidney: a case report
El Kababri, M.	2004	13	Maroc	Clear cell sarcoma of the kidney. A study of 13 cases
Wang, H. M. 2003	2004	1	China	Diagnosis and treatment of extrarenal malignant tumors in children
Ng, A.	2005	1	UK	Clear cell sarcoma: a dilemma on pathological staging and clinical management
Wang, Z.	2005	2	China	Clinicopathological and immunophenotypic characteristics of clear cell sarcoma of the kidney
Zigman, A.	2006	1	USA	Clear cell sarcoma of the kidney with cavo-atrial tumor thrombus: complete resection in a child
Radulescu, V. C.	2008	8	USA	Treatment of recurrent clear cell sarcoma of the kidney with brain metastasis
Hannachi Sassi, S.	2008	2	Tunisie	Clear-cell sarcoma of the kidney. Two pediatric cases
Li, H. X.	2008	1	China	Clear cell sarcoma of kidney with lymph node metastasis around renal pedicle: a case report
Namaoui, R. Y.	2010	1	France	Clear-cell sarcoma of the kidney: about a pediatric case
Sugandhi, N.	2011	1	India	Pediatric clear cell sarcoma of the kidney with cavoatrial thrombus
Stefanowicz, J.	2011	1	Poland	Brain metastases in pediatric patients: characteristics of a patient series and review of the literature
Franco, A.	2011	1	USA	A case of clear cell sarcoma of the kidney
Lal, N.	2011	1	India	Clear cell sarcoma of kidney: A rare entity
Kourti, M.	2012	1	Greece	Rare non-Wilms' tumors in children
Hiradfar, M.	2012	1	Iran	Pediatric clear cell sarcoma of the kidney with atriocaval thrombus
Wang, C. H.	2012	1	China	Clear cell sarcoma of the kidney in child: a case report and review of literature
Hartman Jr, R. J.	2013	1	USA	Incidental capture of rarely diagnosed pediatric tumor: An infant boy with clear cell sarcoma of the kidney
Zekri, W.	2014	4	Egypt	Clear cell sarcoma of the kidney: patients' characteristics and improved outcome in developing countries
Sinha, S.	2014	2	India	Clear cell sarcoma of the kidney: report of two cases
SukdevJadhav, A.	2014	1	India	Clear cell sarcoma of kidney in a neonate
Wang, C. B.	2014	1	China	Clinicopathologic analysis and literature review of clear celI sarcoma of the kidney in children
Hirose, M.	2015	1	Japan	Clear cell sarcoma of the kidney distinguished from synovial sarcoma using genetic analysis: A case report
Kato, M.	2015	1	Japan	Clear cell sarcoma of the kidney with calcification and a novel chromosomal abnormality: A case report
Xu, H. Y.	2015	6	China	Clear cell sarcoma of kidney in children: a clinicopathological analysis of 6 cases
Sheng, Q.	2016	7	China	Clinical treatment and follow-up of 7 children with renal clear cell sarcoma
Weaver, J.	2017	1	USA	Bladder Recurrence of Clear Cell Sarcoma of the Kidney 7 Years After Initial Presentation
Liu, F.	2017	9	China	Clinical analysis of clear cell sarcoma of the kidney in children
Ozdemir, Z. C.	2018	1	Turkey	Renal clear cell sarcoma presenting as a spontaneous renal hematoma: A rare presentation
Wang, G. N.	2018	20	China	Diagnosis and treatment of clear cell sarcoma of the kidney in children
Zhang, H. CH.	2018	1	China	Clear cell sarcoma of kidney: a case report and literature review
Wang, J. H.	2019	7	China	Neoadjuvant transcatheter arterial chemoembolization and systemic chemotherapy for treatment of clear cell sarcoma of the kidney in children
Gao, N. K.	2019	5	China	Report of 5 cases of renal clear cell sarcoma in children
Chen, S.	2020	3	China	Clear cell sarcoma of the kidney in children: a clinopathologic analysis of three cases
Hu, H. M.	2020	10	China	Diagnosis, treatment and prognosis of 10 children with clear cell sarcoma of the kidney in middle and late stage
Lin, J.	2020	3	China	Detection of BCOR and YWHAE-NUTM2B/E genes in children's renal clear cell sarcoma
Wang, X. J.	2020	10	China	Clinicopathological analysis of 10 cases with clear cell sarcoma of the kidney
Dong, J. J.	2021	3	China	Retrospective analysis of outcomes in patients with clear cell sarcoma of the kidney: A tertiary single-institution experience
Friesenbichler, W.	2021	12	Austria	Clear cell sarcoma of the kidney in Austrian children: Long-term survival after relapse
Uchimura, R.	2021	1	Japan	Successful transcatheter arterial embolization to control intratumoral hemorrhage in clear cell sarcoma of the kidney

**Table 2:** Clinical characteristics in the OS group and the Chi-square test between TS and VS.

**Variables**	**Overall**	**Training**	**Validation**	***P***
		**set**	**set**	
	**(*N* = 259)**	**(*N* = 181)**	**(*N* = 78)**	
**Sex**				0.095
Female	76 (29.3%)	47 (26.0%)	29 (37.2%)	
Male	183 (70.7%)	134 (74.0%)	49 (62.8%)	
**Age at initial diagnosis (year)**				0.076
Mean (SD)	2.85 ± 2.19	3.00 ± 2.27	2.50 ± 1.97	
Median [Min, Max]	2.00 [0, 11.0]	2.00 [0, 11.0]	2.00 [0, 9.0]	
**Laterality**				0.163
Left	134 (51.7%)	88 (48.6%)	46 (59.0%)	
Right	125 (48.3%)	93 (51.4%)	32 (41.0%)	
**Stage**				
I	78 (30.2%)	99 (54.7%)	39 (50.0%)	0.576
II	60 (23.2%)			
III	88 (33.9%)	82 (45.3%)	39 (50.0%)	
IV	33 (12.7%)			
**Radiotherapy**				
Yes	210 (81.1%)	147 (81.2%)	63 (80.8%)	0.999
No	49 (18.9%)	34 (18.8%)	15 (19.2%)	
**Chemotherapy**				
Yes	249 (96.1%)	175 (96.7%)	74 (94.9%)	0.494
No	10 (3.9%)	6 (3.3%)	4 (5.1%)	
**Survival time (month)**				
Mean (SD)	73.4 ± 80.2			
Median [Min, Max]	38.0 [1, 344]			
**Outcome**				
Alive	206 (79.5%)			
Dead	53 (20.5%)			

The distribution of stage I, II, III, and IV tumors were 30.2, 23.2, 33.9, and 12.7% respectively. Bone, lung, liver, and soft tissue made up the top four DM locations, while brain, spleen, chest wall, and other locations were less common. The most common initial clinical symptoms were abdominal mass (234/259) and haematuria (68/259), followed by abdominal pain (19/259), hypertension (15/259), abdominal distension (14/259), and fever (11/259). A very small number of patients also presented with gastrointestinal symptoms such as nausea, vomiting, and constipation, as well as bone pain due to tumor bone metastases.

All patients included in the OS group underwent surgery, of which 210 (81.8%) received radiotherapy and all of them received postoperative radiotherapy. A total of 249 (96.1%) patients received chemotherapy. There were 206 survivors and 53 deaths, including 14 deaths in 138 stage I–II patients, 17 deaths in 88 stage III patients, and 22 deaths in 33 stage IV patients, indicating that most deaths occurred in patients with DM. The survival time of these patients ranged from 1 month to 28.7 years, with a mean of 73.4 months.

### Nomogram construction and validation in OS group

The OS group was randomly divided into TS (*n* = 181) and VS (*n* = 78) groups. The six potential predictors for predicting the OS of CCSK patients were not statistically significantly different in the two groups (Table 2). The univariate and multivariate Cox regression showed radiotherapy (*P* < 0.001), chemotherapy (*P* = 0.001), and stage (*P* < 0.001) were independent OS-related factors of CCSK patients and they were included in the nomogram. Stage III–IV (HR = 3.630) were risk factors and radiotherapy (HR = 0.300) and chemotherapy (HR = 0.104) were protective factors (Table 3).

**Table 3:** Univariate and multivariate Cox regression in OS group.

**Factors**	**Univariate Cox regression**			**Multivariable Cox regression**		
	**HR**	**95CI**	***P***	**HR**	**95CI**	***P***
**Sex**
Female	Reference					
Male	1.113	0.507–2.447	0.789			
**Age**						
<2	Reference					
2–4	0.939	0.439–2.005	0.870			
>4	1.106	0.445–2.750	0.829			
**Laterality**
Left	Reference					
Right	1.893	0.947–3.786	0.071			
**Stage**
I, II	Reference			Reference		
III, IV	3.182	1.565–6.470	0.001	3.630	1.775–7.423	<0.001
**Radiotherapy**
No	Reference			Reference		
Yes	0.292	0.148–0.578	<0.001	0.300	0.145–0.622	0.001
**Chemotherapy**
No	Reference			Reference		
Yes	0.092	0.035–0.240	<0.001	0.104	0.036–0.302	<0.001

CI, confidence interval; HR, Hazard ratio.

**Figure 2:**
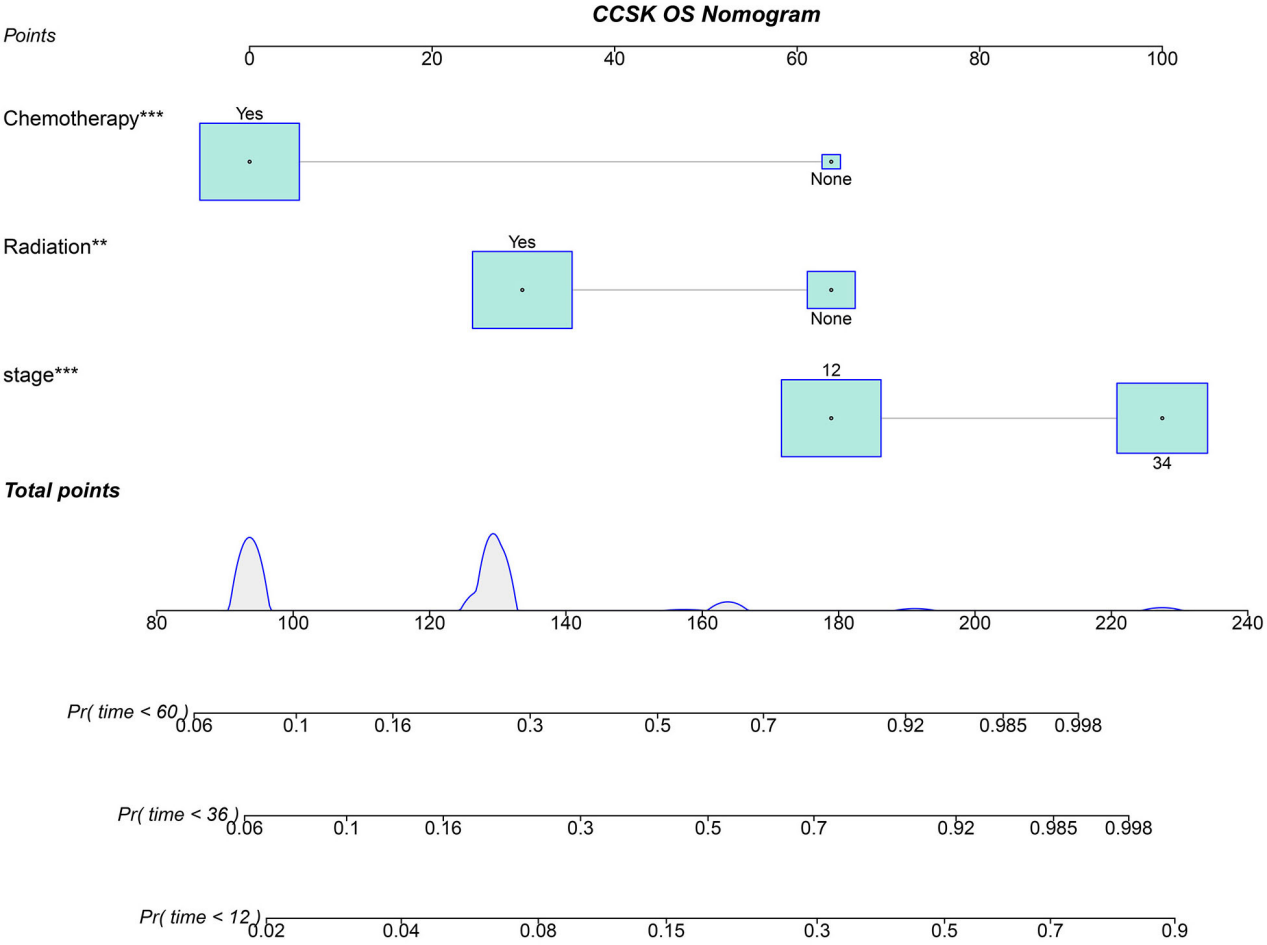
Nomogram for predicting the OS of CCSK for the 12, 24, and 36 months. 12, stage I and II; 34, stage III and IV; Pr, the probability that the OS of a patient is <12, 36, and 60 months. *, is an automatically generated label for statistical significance when plotted figure by the R software. The more * appeared, the more statistically significant.

Based on three independent predictors, the nomogram for predicting the 12, 36, and 60 months OS of CCSK patients with an initial diagnosis was constructed (Figure 2). ^*^ in Figure 2 is an automatically generated label for statistical significance when plotting Figure 2 by the R software. The more ^*^ appeared, the more statistically significant. The first step of using a nomogram is to draw a straight line between the specified point of each factor and the points line to get the corresponding score. Second, the sum of the specified point score plotted on the Pr line represents the probability that the OS is <12, 36, and 60 months.

**Figure 3:**
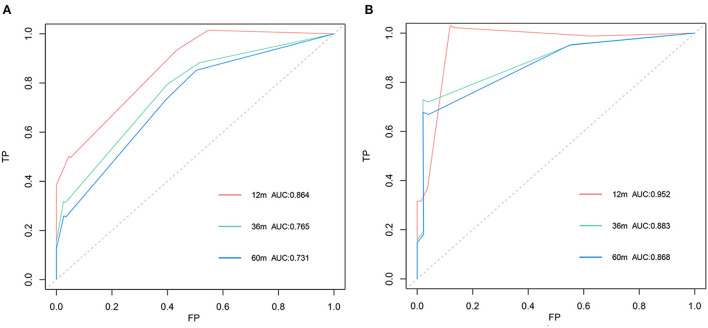
Time–dependent ROC in the training set **(A)** and in the validation set **(B)**.

**Figure 4:**
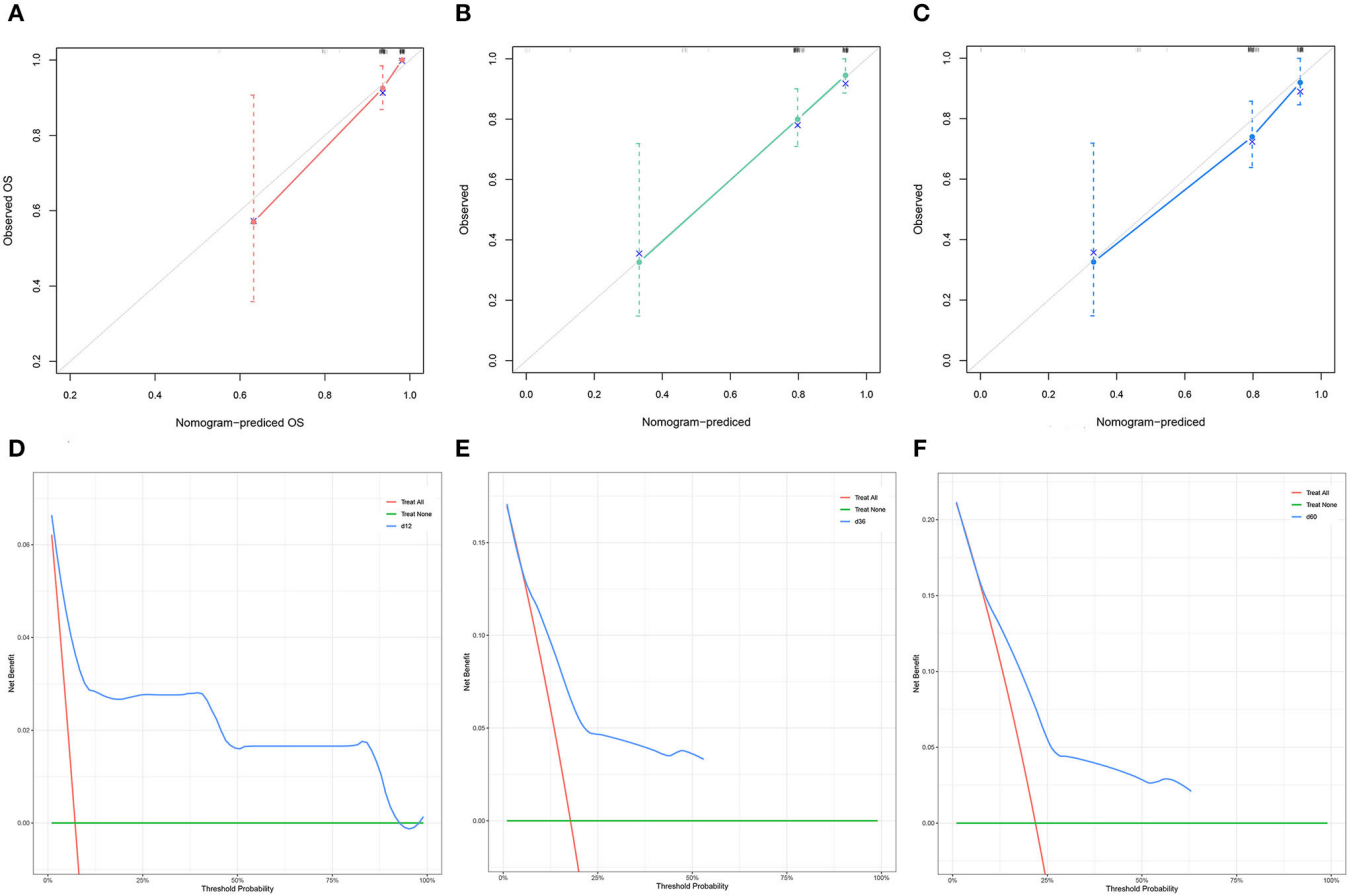
The calibration curves of the nomogram for the 12 **(A)**, 36 **(B)**, and 60 **(C)** months in the training set. The decision curve analysis of the nomogram for the 12 **(D)**, 36 **(E)**, and 60 months **(F)** in the training set.

**Figure 5:**
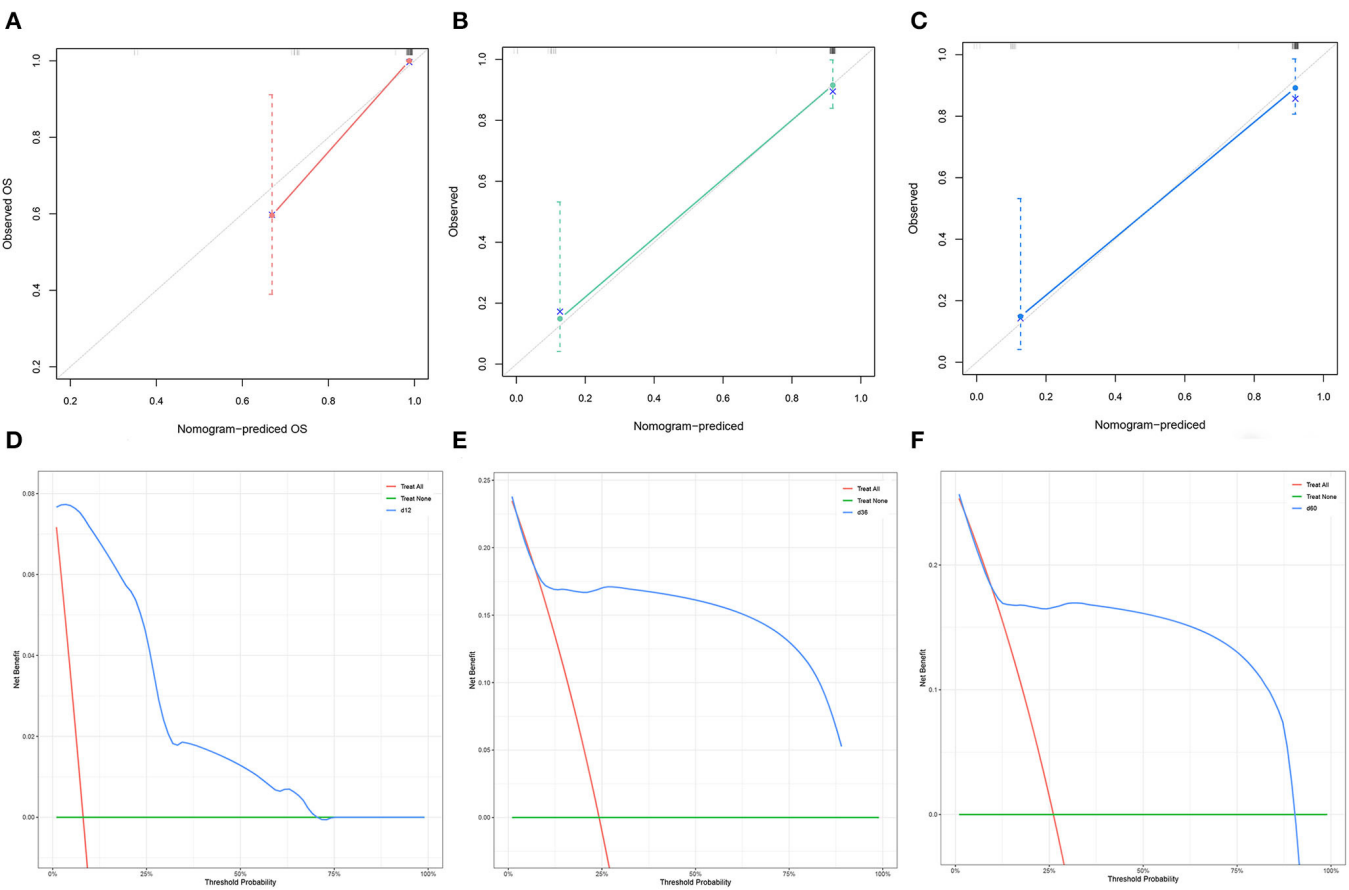
The calibration curves of the nomogram for the 12 **(A)**, 36 **(B)**, and 60 **(C)** months in the validation set. The decision curve analysis of the nomogram for the 12 **(D)**, 36 **(E)**, and 60 months **(F)** in the validation set.

The C-index of the nomogram was 0.754 for TS and 0.878 for VS. The AUC of the time-dependent ROC curves at 12, 24, and 36 months all showed a good performance with 0.864, 0.765, and 0.731 in the TS and 0.952, 0.883, and 0.868 in the VS (Figure 3), which implied good discrimination of the model (19). In the calibration curves which assess the consistency between nomogram prediction and actual observation, the x-axis represented the nomogram-projected risk of CCSK and the y-axis represented the observed risk of CCSK. The diagonal line represented the perfect prediction. The solid line represented the performance of the nomogram, which is closer to the diagonal line to indicate a better prediction. Our calibration curves showed high consistency between observed and nomogram-predicted for TS (Figures 4A–C) and VS (Figures 5A–C) at 12, 36, and 60 months. In the DCA curves, the x-axis represented the diagnostic threshold and the y-axis represented the net benefit. The green line represented the net benefit of the assumption that all CCSK patients were dead. The pink line represented the net benefit of assuming no death. The blue line represented the net benefit of our nomogram. The farther the blue line is from the pink and green lines, the greater the net benefit that can be obtained by using the nomogram. The DCA curve showed that the nomogram can be used to obtain a good net benefit in a wide range (Figures 4D–F, 5D–F).

**Figure 6:**
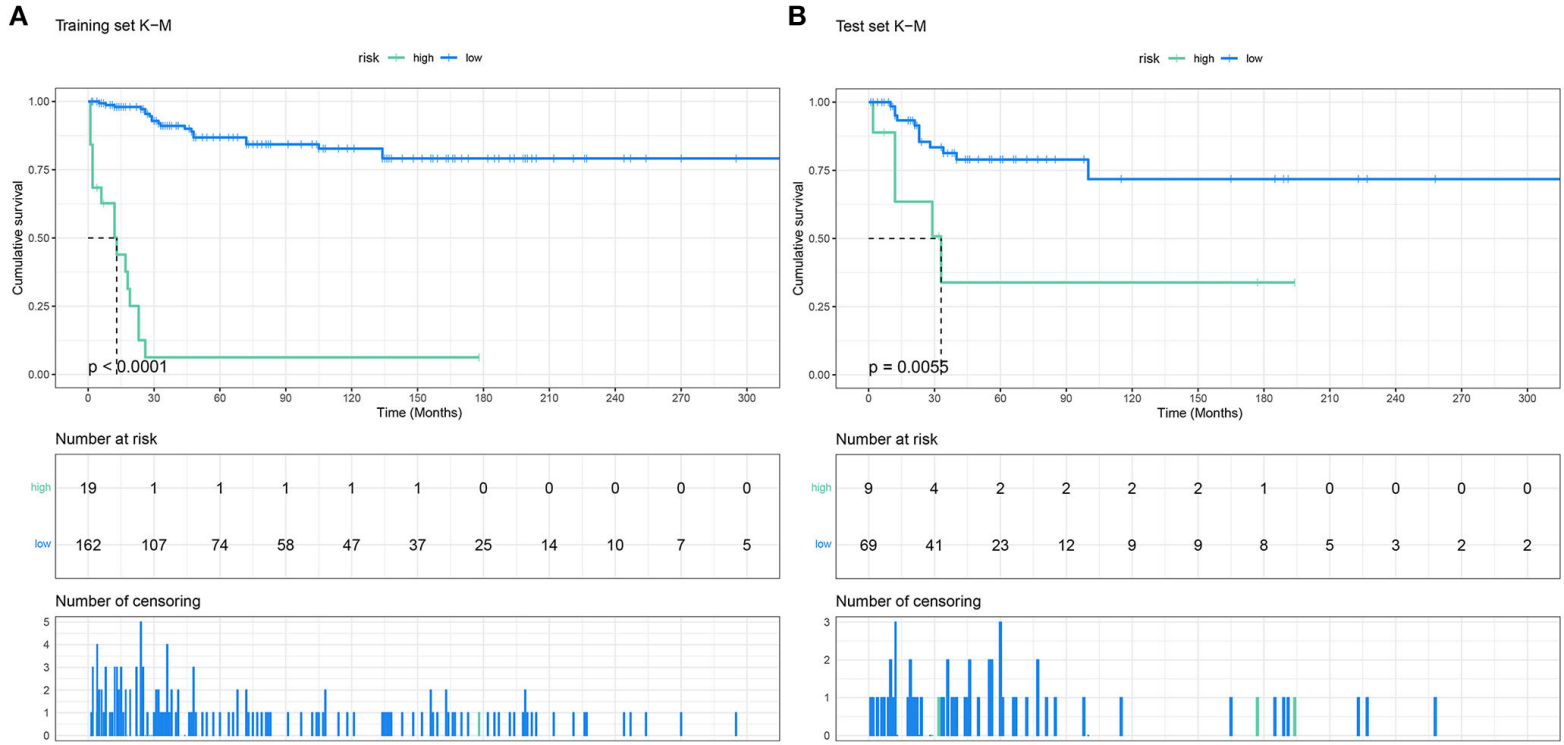
The Kaplan–Meier survival curves of the patients in the training set **(A)** and in the validation set **(B)**.

The points of the predictors included in the nomogram are as follows: Chemotherapy Yes = 0, None = 64; Radiotherapy Yes = 30, None = 64; stage I–II = 64, III–IV = 100. After applying the cut-off value to patients' Nomo-score with X-tail software, two risk stratification groups were generated: 94 ≤ low risk ≤ 158; 164 ≤ high risk ≤ 228. The K–M survival curves of the two risk groups (Figure 6) with log-rank test (*P* < 0.001) showed obvious grading ability.

### Relapse characteristics and related factors in the relapse group

Although details of 162 patients were obtained from online databases, nine patients had not reported their relapse information. Hence, the relapse group included 153 patients, of which 60 had relapsed. Information on relapse group is shown in Table 4. After the initial diagnosis, 103 patients were treated with postoperative chemotherapy, 42 with preoperative and postoperative chemotherapy, and 8 without chemotherapy. Regional differences in chemotherapy regimens were significant, with 75 of the 145 chemotherapy-treated patients using the NWTS-5 or Children's Oncology Group (COG) regimen, 17 patients using the SIOP regimen, and the remaining patients reporting only the type of chemotherapy drug used without specifying the regimen.

**Table 4:** Clinical characteristics in the relapse group.

**Factors**	**Overall**	**Non-relapse**	**Relapse**
	**(*N* = 153)**	**(*N* = 93)**	**(*N* = 60)**
**Sex**
Female	46 (30.1%)	31 (33.3%)	15 (25.0%)
Male	107 (69.9%)	62 (66.7%)	45 (75.0%)
**Age at initial diagnosis** (year)
Mean (SD)	2.91 ± 2.12	2.94± 2.26	2.84± 1.90
Median [Min, Max]	2.00 [0, 11.0]	2.3 [0.3, 11.0]	2.3 [1, 7.3]
**Laterality**
Left	77 (50.3%)	44 (47.3%)	33 (55.0%)
Right	76 (49.7%)	49 (52.7%)	27 (45.0%)
**Stage**
I	47 (30.7%)	37 (39.8%)	10 (16.7%)
II	27 (17.7%)	18 (19.4%)	9 (15.0%)
III	53 (34.6%)	27 (29.0%)	26 (43.3%)
IV	26 (17.0%)	11 (11.8%)	15 (25.0%)
**Radiotherapy**
Yes	113 (73.9%)	68 (73.1%)	45 (75.0%)
No	40 (26.1%)	25 (26.9%)	15 (25.0%)
**Surgery/chemotherapy sequence**
Postoperative chemotherapy	103 (67.3%)	65 (69.9%)	38 (63.3%)
Both^*^	42 (27.5%)	27 (29.0%)	15 (25.0%)
No chemotherapy	8 (5.2%)	1 (1.1%)	7 (11.7%)
**Use of chemotherapeutic drugs**
Actinomycin	54 (35.3%)	28 (30.1%)	26 (43.3%)
Vincristine	134 (87.6%)	87 (93.5%)	47 (78.3%)
Cyclophosphamide	104 (68.0%)	70 (75.3%)	34 (56.7%)
Doxorubicin	123 (80.4%)	81 (87.1%)	42 (70.0%)
Etoposide	100 (65.4%)	67 (72.0%)	33 (55.0%)
Ifosfamide	19 (12.4%)	13 (14.0%)	6 (10.0%)
Carboplatin	24 (15.7%)	17 (18.3%)	7 (11.7%)
**Relapse time** (month)
Mean (SD)			32.7 ± 35.7
Median [Min, Max]			22.0 [1, 202]
**Location of relapse**
Local			8
Bone			31
Brain			25
Lung			18
Liver			2
Soft tissue			6
Other (Mediastinum, Bladder, Orbit)			10
**Combined relapses**
Yes			18 (30.0%)
No			42 (70.0%)
**Outcome**
Alive	118 (75.2%)	90 (96.8%)	25 (41.7%)
Dead	35 (24.8%)	3 (3.2%)	35 (58.3%)

* Both: Preoperative and postoperative chemotherapy.

In 60 patients with relapse, the ratio of male to female is 3:1. The average age of initial diagnosis was 2.84 ± 1.90 years old. Tumor primary renal tendency was homogeneous, left: right = 1.22:1. 53 (88.3%) patients were distant relapses. The most common locations of relapse were bone, brain, lung, local, liver, and soft tissue. The uncommon relapse sites included bladder (20, 21), orbit (2), soft tissue (22), and chest wall (23). Eighteen patients had combined relapses, of which three patients had multiple metastases all over the body. The most common location of combined relapses was bone and lung (4/18), bone and brain (3/18), and local and bone (3/18). About 38 (63.3%) relapsed patients died. The time from the first diagnosis to relapse ranged from 1 to 202 months, with a mean of 32.7 months. The 5-years OS was 22.9%.

The multivariate cox regression analysis results (Table 5) for relapse showed that: (i) stage III and IV were significant relapse-related risk factors; (ii) Chemotherapy was a relapse-related effective therapeutic. No chemotherapy (HR = 5.005, *P* < 0.001) would significantly increase the risk of relapse; Both preoperative and postoperative chemotherapy (HR = 0.568 *P* = 0.077) would better prevent relapse compared to postoperative chemotherapy only (Reference); (iii) The relapse rate of patients with radiotherapy decreased by 2.3% (39.8 and 37.5%) compared with those without radiotherapy. But the univariate multifactorial cox regression was not statistically significant (*P* > 0.05). Given the small sample of this group, we did not model the prediction of relapse.

**Table 5:** Univariate and multivariate Cox regression in relapse group.

**Factors**	**Univariate Cox regression**			**Multivariable Cox regression**		
	**HR**	**95CI**	***P***	**HR**	**95CI**	***P***
**Sex**
Female	Reference					
Male	1.303	0.725–2.339	0.376			
**Age**
Mean (SD)	0.996	0.985–1.007	0.472			
**Laterality**
Left	Reference					
Right	0.772	0.464–1.285	0.319			
**Stage**
I	Reference			Reference		
II	1.704	0.691–4.201	0.247	2.231	0.973–6.294	0.087
III	2.473	1.192–5.131	0.015	3.270	1.804–8.381	0.001
IV	5.800	2.566–13.113	<0.001	7.011	2.946–16.688	<0.001
**Radiotherapy**
Yes	Reference					
None	1.702	0.943–3.073	0.078			
**Surgery/chemotherapy sequence**
Postoperative chemotherapy	Reference			Reference		
Both^*^	0.895	0.492–1.630	0.718	0.568	0.303–1.064	0.077
No chemotherapy	5.647	2.479–12.870	<0.001	5.005	2.003–12.510	<0.001

* Both: Preoperative and postoperative chemotherapy; CI, confidence interval; HR, Hazard ratio.

**Figure 7:**
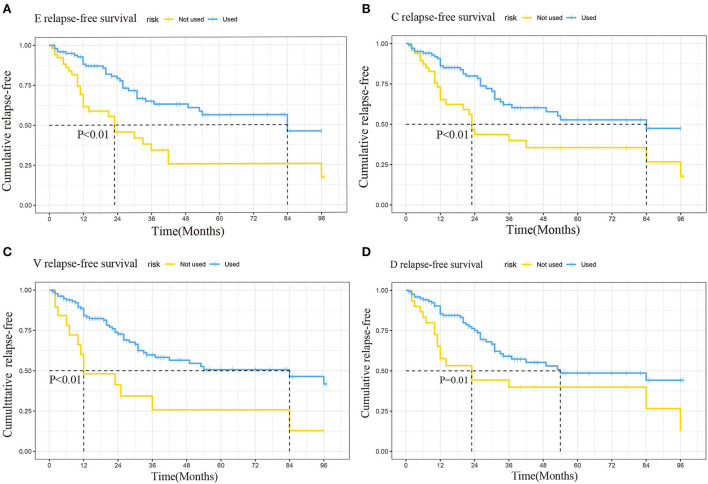
Relapse-free survival curves of etoposide **(A)**, cyclophosphamide **(B)**, vincristine **(C)**, doxorubicin **(D)**.

The log-rank test of the seven chemotherapeutic drugs showed that etoposide (E), cyclophosphamide (C), vincristine (V), and doxorubicin (D) (all *P* < 0.05) had a significant difference in preventing relapse (Table 6). Relapse-free survival curves of EVCD are shown in Figure 7.

**Table 6:** Log-rank test of chemotherapeutic drugs in the relapse group.

**Use of chemotherapeutic drugs**	**Relapse group (*N* = 153)**	***P***
**Actinomycin**		0.1
No	99 (64.7%)	
Yes	54 (35.3%)	
**Vincristine**		<0.001
No	19 (12.4%)	
Yes	134 (87.6%)	
**Cyclophosphamide**		0.002
None	49 (32.0%)	
Yes	104 (68.0%)	
**Doxorubicin**		0.01
No	30 (19.6%)	
Yes	123 (80.4%)	
**Etoposide**		<0.001
No	53 (34.6%)	
Yes	100 (65.4%)	
**Ifosfamide**		0.2
No	134 (87.6%)	
Yes	19 (12.4%)	
**Carboplatin**		0.3
No	129 (84.3%)	
Yes	24 (15.7%)	

## Discussion

### Nomogram in OS group

The first purpose of our study was to obtain a large sample to explore the predictors of OS and establish an easy-to-use nomogram to fill the gap in the accurate prediction of CCSK prognosis. Clinical research of CCSK in recent years showed that the combined treatment of surgical resection + chemotherapy + radiotherapy has a 5-years RFS of 72.9–85% and a 5-years OS of 74.5–88% (4–8, 24). It can be seen that with the optimization of diagnosis and treatment guidelines, the survival rate had a good performance, but some patients still have poor prognoses. Therefore, it is necessary to establish a tool for numerical prediction of survival probability. Our nomogram was designed for patients with an initial diagnosis. It can predict the 1-, 3-, and 5-years OS of patients based on chemotherapy, radiotherapy, and stage.

The stage is the most powerful predictor of OS in our study. It is the most significant risk factor for survival which has been widely recognized (2, 25, 26). The Cox regression results showed that stages III and IV were significant risk factors for the prognosis of CCSK. The majority of patients with CCSK are in stages I–III, which may be the main reason for the fair prognosis of this disease. The clinicians should proceed to improve prognosis by avoiding misdiagnosis to prevent patients' stage progression (27, 28).

There is no doubt that chemotherapy is a protective factor for patient survival. After the use of intensive chemotherapy in recent decades, the prognosis of patients with stage I–III was optimistic, while that of patients with stage IV was still pessimistic (29). The inability of stage IV patients to benefit from current treatment poses a difficult problem. The treatment of stage IV with high-dose chemotherapy as the mainstay is approaching the maximum tolerance intensity of drugs, which highlights the urgent need for new treatment strategies (6). Future research should focus on international cooperation in developing new targeted therapies based on CCSK biological characteristics.

Radiotherapy was also a protective factor. However, due to the sequelae and iatrogenic complications of radiotherapy, whether it should be applied to stage I patients has always been a controversial issue (30). The chi-squared test in our study showed no significant difference in OS with radiotherapy or not in stage I patients. In the current treatment regimen, COG-AREN0321 and SIOP UMBRELLA advocates that all patients should receive 10.8 Gy radiotherapy. A follow-up study of 53 stage I patients reported that the cancer-specific survival rate was 100% in seven children who did not receive radiotherapy (31). So it is of great practical value that COG's subsequent diagnostic specifications further refine the radiotherapy regimen for patients in stage I.

The correlation between these three predictors (stage, chemotherapy, and radiotherapy) and the OS of CCSK has been widely recognized. The significance of our nomogram is that it is the first tool to quantify and visualize this correlation concretely. In addition, our nomogram divides patients into two risk groups through risk stratification. The OS of patients with nomogram scores in the high-risk range may be shorter. Clinicians can guide the individualized monitoring of patients during follow-up.

### Relapse-related factors in the relapse group

Rarity represents the greatest barrier to advances in the diagnosis and treatment of CCSK. There have been several follow-up clinical studies on CCSK relapse (5, 16–18, 32). Most of them were single-center observations with small samples. There is only a large cohort of 37 out of 237 patients with relapse (5). The second aim of this study was to obtain a large sample to observe the consistency of the relapse characteristics we obtained with those reported in previous studies, and to explore the relapse-related factors.

The statistics of relapse characteristics in our study are in good agreement with the results of several existing clinical studies, which indicate that medicine has a relatively unified understanding of the characteristic of relapse of CCSK at present (5, 17, 32). Differentially, the largest clinical study of 237 CCSK patients with 37 relapses to date by Gooskens et al. (5) has shown that patients younger than 12 months at the time of initial diagnosis are more likely to relapse. However, our study did not observe this tendency to relapse in young children. In the relapse group, of the 34 patients who were <12 months old at the initial diagnosis, 14 relapsed and 20 did not. So, this tendency needs to be validated with a larger sample. It is worth mentioning that, unlike the small proportion of patients with distant metastases at initial diagnosis (33–35), almost all relapses are metastatic (36). The most common location of relapse involves the brain, which is distinct from the most common location of DM being bone, lung, and liver (26). Gooskens et al. (5) believes the brain is a safe haven for CCSK tumor cells during chemotherapy. Her study showed brain relapse in 7% of patients, so it is recommended that brain MRI should be used in all patients during follow-up. In our study, 20 (80%) of the 25 patients with brain relapse were in stage III or IV at the time of initial diagnosis. Based on this we believe that differentiating the frequency of brain MRI reexamination between stage I–II and stage III–IV in further refinement of follow-up diagnostic specifications may provide additional economic value.

There is currently no consistent standard of treatment for patients with relapse. In the study of 37 relapsed patients by Gooskens et al. (5), complete postoperative local control with chemotherapy and/or radiotherapy was shown to improve the survival of some patients. However, its 5-years EFS of 18% and 5-years OS of 26% after relapse still showed a pessimistic prognosis overall. The study also reported that a total of 24 patients with relapse received high-dose chemotherapy and autologous bone marrow transplantation, 12 of whom survived an average of 52 months of follow-up. Radulescu (37) reported 8 patients with brain relapse. In addition to chemotherapy with ifosfamide, carboplatin, and etoposide, four patients received Stem Cell Transplantation (SCT). Two patients died and two survived 29 and 71 months after relapse. But the number of SCT-related samples was too small to draw an accurate conclusion about its therapeutic effect. It followed that relapsed patients face the same dilemma as patients in stage IV: existing treatments cannot effectively enhance their survival. In this case, exploring how to avoid relapse is valuable. This prompted us to explore which factors are associated with relapse and which treatments avoid relapse. In the relapse group, we identified three related factors: stage, surgery/chemotherapy sequence, and chemotherapy drugs ECVD. Radiotherapy had no significant contribution to preventing relapse.

The stage of the patient is a significant risk factor for relapse, which has been confirmed in previous studies (2). For chemotherapy, the patient information in the relapse group contained surgery/chemotherapy sequence compared to the OS group, so we refined the chemotherapy variable in the Cox regression analysis in the relapse group. We concluded that both preoperative and postoperative chemotherapies were more effective in preventing relapse than only postoperative chemotherapy, which is consistent with the proposition of SIOP for preoperative treatment (6). In the current treatment regimens, COG is based on immediate nephrectomy, while SIOP is characterized by preoperative chemotherapy. SIOP trials demonstrated the advantages of preoperative chemotherapy in reducing regional lymph node metastasis and intraoperative tumor rupture (4). Radical nephrectomy after preoperative chemotherapy ensures the possibility of complete resection of the affected kidney and tumor in patients, thus improving the cure rate in children at an advanced stage (38, 39). And the UK Children's Cancer Study Group randomized trial (40) showed a significant improvement in stage distribution in patients with non-metastatic nephroblastoma who had undergone 6 weeks of chemotherapy followed by surgery compared with those with immediate surgery. In addition, we compared the chemotherapy drug in preventing relapse by Log-rank test. The chemotherapy drug with a statistically significant difference in preventing relapse was ECVD, which is consistent with the stage I–IV chemotherapy regimen of NWTS-5, and it can be considered that they have a good effect on preventing relapse. The 19% relapse rate in the NWTS-5 trial was lower than in previous studies (36).

The biggest difference of factors between the two groups was that there was no correlation between radiotherapy and relapse but there was a correlation with OS (31). This conclusion was put forward for the first time with fewer relevant supporting studies. The results of the SIOP study (4) showed that of the 85 patients who received radiotherapy, recurrence happened in 13, while it relapsed in 17 of 92 patients treated without radiotherapy. Since they did not undergo multi-factor regression, it cannot fundamentally support our conclusion.

The rarity of the condition limits advances in the diagnosis and treatment of CCKS. The prognosis of patients with relapse is still pessimistic, on the one hand, to improve the further exploration of effective prevention of relapse, and on the other hand, to develop new therapeutic strategies based on specific molecular and genetic variations. Therefore, it is particularly important to strengthen international cooperation studies and trials.

Our study has several shortcomings. First, the treatment details and multidisciplinary therapy (e.g., the opportunity for surgery, radiotherapy and chemotherapy, chemotherapy regimens, etc.) in the SEER database are not provided. Including these factors will increase the predictive ability of our nomogram. A study that records more cases of potential factors is needed to establish a relapse prediction model. Second, the descriptions of the regional and the distal lymph nodes in the reported cases vary greatly, and cannot be summarized into a unified variable to be included in the study. The addition of these details can improve the prediction ability of nomograms to provide clearer clinical guidance.

In the OS group, it was concluded that stage, chemotherapy, and radiotherapy were independent predictors of OS in CCSK. The constructed nomogram was validated as an effective tool to accurately quantify the OS of CCSK. This fills the research gap in predicting the prognosis of CCSK and is helpful for clinical decision-making. And the chi-squared test showed no significant difference in OS with radiotherapy or not in stage I patients. This conclusion could be an effective basis for improving the primary treatment program. In the relapse group, stage, surgery/chemotherapy sequence, and chemotherapy ECVD were concluded to be relapse-related factors. Radiotherapy did not significantly contribute to preventing relapse. These conclusions provide information for changing supporting treatment guidelines which have the potential to improve OS.

## Data availability statement

The original contributions presented in the study are included in the article/supplementary material, further inquiries can be directed to the corresponding author.

## Author contributions

YZ conceived the study, did the statistical analysis, and wrote the article. QC and CM were involved in data acquisition. J-jD and YM contributed to designing the project and reviewing the manuscript. All authors contributed to the article and approved the submitted version.

## Conflict of interest

The authors declare that the research was conducted in the absence of any commercial or financial relationships that could be construed as a potential conflict of interest.

## Publisher's note

All claims expressed in this article are solely those of the authors and do not necessarily represent those of their affiliated organizations, or those of the publisher, the editors and the reviewers. Any product that may be evaluated in this article, or claim that may be made by its manufacturer, is not guaranteed or endorsed by the publisher.

## Abbreviations

CCSK: clear cell sarcoma of the kidney
SEER, surveillance, epidemiology: and end results
NWTSG: North American national Wilms ‘tumor study group
NWTS-5: NWTSG trials 5
COG: children's oncology group
SIOP: European UMBRELLA international society of pediatric oncology
OS: overall survival
DM: distal metastasis
WT: Wilms ‘tumor
EGFR: epidermal growth factor receptor
CNKI: China knowledge network
TS: training set
VS: validation set
E: etoposide
C: cyclophosphamide
V: vincristine
D: doxorubicin
C-index: concordance-index
ROC: receiver operating characteristic
DCA: decision curve analysis
K–M: Kaplan–Meier
UKW3: UK Wilms tumor trial 3
HR: hazard ratio
CI: confidence interval.
